# Rate of Social Isolation by Geographic Location Among Older Adults: AAA LongROAD Study

**DOI:** 10.3389/fpubh.2021.791683

**Published:** 2021-12-10

**Authors:** Laura Lynch, Thelma J. Mielenz, Guohua Li, David W. Eby, Lisa J. Molnar, Marian E. Betz, Carolyn DiGuiseppi, Linda L. Hill, Vanya Jones, David Strogatz

**Affiliations:** ^1^Department of Epidemiology, Mailman School of Public Health, Columbia University Irving Medical Center, New York, NY, United States; ^2^Department of Anesthesiology, Vagelos College of Physicians and Surgeons, Columbia University Irving Medical Center, New York, NY, United States; ^3^Transportation Research Institute, University of Michigan, Ann Arbor, MI, United States; ^4^Department of Emergency Medicine, School of Medicine, University of Colorado, Aurora, CO, United States; ^5^Department of Epidemiology, Colorado School of Public Health, University of Colorado Denver, Aurora, CO, United States; ^6^Department of Family Medicine and Public Health, School of Medicine, University of California, San Diego, San Diego, CA, United States; ^7^Department of Health, Behavior and Society, Bloomberg School of Public Health, Johns Hopkins University, Baltimore, MD, United States; ^8^Bassett Research Institute, Mary Imogene Bassett Hospital, Cooperstown, NY, United States

**Keywords:** geography, modifiable risk, driving, social isolation, older adults

## Abstract

**Introduction:** Social isolation is a modifiable risk factor for negative health outcomes among older adults. This work assessed the relationship between geography (i.e., urban vs. non-urban residence) and social isolation in a cohort of older drivers.

**Methods:** The AAA LongROAD cohort with 2,989 older adult drivers from across the country were included. Social isolation was measured at baseline and at two subsequent annual follow-ups using PROMIS v2.0 Social Isolation 4a. The effect of geographic location with social isolation was assessed through with multivariable regression using a generalized estimating equation model.

**Results:** The rate of social isolation in urban areas was 21% lower (adjusted RR 0.79, 95% CI 0.46, 1.36) compared to non-urban areas after adjusting for covariates, though not significant.

**Discussion:** Social isolation is a predictor of poor health outcomes and geographic considerations have been lacking in the literature. The panel data in this analysis provides more evidence for causality though the under-representation of non-urban areas potentially reduces the power for the results.

**Conclusions:** It is important to understand the needs and risk of social isolation in various geographic settings to ensure resources and interventions are appropriately modified for a greater public health impact.

## Introduction

The older adult population in the United States (US) is steadily growing, with adults over 65 projected to outnumber those under 18 for the first time in US history (1, 2). The rapidly growing older adult population is affected by age-related functional decline, increased prevalence of acute and chronic diseases, and the prospect of a loss of independence through driving cessation. The latter is associated with significant health decline including worsening physical function and increased depression (1, 3–5). It was founded that a modifiable risk factor associated with mortality and increased medical costs is social isolation (6–8). A national assessment found that 24% of older adults in the United States are socially isolated (6). The construct of social isolation assesses social network, community connections and participation in society (5, 6, 8, 9).

We assessed if geography was associated with social isolation with a more precise measure of social isolation to build on this conversation in the literature. Social isolation is a more objective and comprehensive domain compared to loneliness. Along with mortality, social isolation is associated with increased medical costs (7, 8).

To date there has not been consensus on whether social isolation varies by geography. A study measuring prevalence of social isolation found no difference between urban and non-urban areas in the adjusted model where social isolation was measured as physical separation (6). A subsequent cross-sectional study more broadly defining social isolation by rurality found that urban residents had fewer close relatives and friends, and were less able to rely on them than residents of non-metropolitan areas (10). Interestingly, residents in the least populated and most isolated rural places had similar levels of loneliness to their urban counterparts, despite reporting more social connections.

Other research has shown that older adults utilize health care services more than younger patients, and particularly for older adults experiencing loneliness, interactions with the health system can be very important for social and physical health (11). Rural dwelling individuals also have been shown to have poorer access to healthcare, which may be exacerbated by the loss of the ability to drive with advanced age, particularly in settings with poor public transportation infrastructure more common in non-urban settings (12, 13). This creates a challenging situation for older drivers who may be experiencing loneliness or isolation but have a reduced ability to seek care.

Using the AAA Longitudinal Research on Aging Drivers (AAA LongROAD) cohort, we assessed the rate of social isolation among older adults using precise measures normed to the US average. The Patient-Reported Outcomes Measurement Information System (PROMIS) measure for social isolation is a concise and validated measure developed by the National Institutes of Health. It was derived from a loneliness scale which is more focused on the feeling of distress that is associated with social isolation vs. loneliness (14, 15). We hypothesized an increased rate of social isolation with a less pronounced change in more rural areas.

## Methods

The study population is from the AAA LongROAD prospective cohort study. Participants ranged from 65 to 79 years old at baseline and were recruited in one of five sites across the US: Ann Arbor, MI, Baltimore, MD, Cooperstown, NY, Denver, CO, and San Diego, CA. To be included in the study, participants had to reside in one of the above-referenced locations for 80% of the year, have a valid driver's license and have no major cognitive deficits. A total of 2,990 were enrolled in the cohort; more detailed methods are described in the LongROAD methods paper (2). Because we are assessing population averages, participants needed at least one measure of social isolation across the three time intervals (baseline 7/7/2015 to second year follow-up 6/8/2019); one participant did not meet this criterion and was excluded. The study population for this analysis was 2,989.

The self-reported PROMIS v2.0 Social Isolation 4a data was collected at baseline and annually (16). This measure for social isolation is derived from the following four questions: “I feel left out,” “I feel that people barely know me,” “I feel isolated from others” and “I feel that people are around me but not with me” (15, 17). Participants reported the degree to which they agreed with the statements, using Likert response categories which are summed and converted to a standardized T-score. The continuous T-score is normed to the US Census population with the mean being a T-score of 50 and a T-score of 40 being one standard deviation less isolated than the population mean.

Geography based on address of primary residence was dichotomized to urban and non-urban using Rural Urban Commuting Area (RUCA) codes. The original ten RUCA codes based on census tract, population density, and commuting were categorized into urban (1, 1.1), suburban (1.3, 2.2), and rural (>2.2) for the LongROAD study. For this analysis, the categories were dichotomized into urban and non-urban, the latter including the suburban population due to the small sample size. Potential confounders were age group, gender (male/female), race/ethnicity, marital status, education, measures of health and driving exposure (18). Marriage was categorized for the model and included as a dichotomous variable: married or live-in partner; divorced, separated, never married or widowed. Race/ethnicity was analyzed using the following categories: White Non-Hispanic (85.9%), Black Non-Hispanic, Asian, Hispanic and other non-Hispanic. Education categories were advanced degrees (41.6%), Bachelor's; some college, Associates' or vocational; and high school or less. Race/ethnicity, education and miles driven per week were included in the model as dummy variables. Physical status was measured with PROMIS SF v.1.0- Physical Function 4a dichotomized at the mean; scores <50 indicated worse than average physical function. Vision and hearing were dichotomized self-reported measures (Very Good to Excellent vs. Poor to Good) (10, 19). Social role (PROMIS Item Bank v2.0- Ability to Participate in Social Roles and Activities) and depressive symptoms (PROMIS Short Form v1 Depression 4a) both produce T-scores, with higher scores indicating increased depressive symptoms and increased ability to participate in social roles (20). Life satisfaction was measured on a scale from one to five with five representing the highest satisfaction (21). Driving frequency was self-reported miles driven per week. This variables was included because driving status is associated with social isolation (3, 8, 22).

Baseline characteristics were stratified by exposure status, i.e., urban or non-urban, and compared using χ^2^ and *T*-tests. To account for correlation from repeated measures within individuals, a generalized estimating equation (GEE) was used to assess the rate of social isolation using an identity link, Gaussian distribution and unstructured correlation structure. Life satisfaction and social role were highly correlated with one another and therefore excluded from modeling to increase the model's precision. Their collinearity approached 0.4. Time-varying covariates included in the model were age, race/ethnicity, marital status, education, depression, physical function, hearing and driving exposure.

## Results

The population in this analysis was predominantly white (85.5%), married (66.0%) and held advanced degrees (40.8%) (Table 1). Below average depression (93.6%) and social isolation (mean 42.0, SD 6.9) were reported as well as higher levels of life satisfaction (mean 3.9, SD 0.6) (Table 1). Physically this cohort self-reported very good vision (67.1%) and hearing (88.3%) with 54.2% scoring better than average on physical function (Table 1).

**Table 1:** Baseline characteristics by geographic status = 2,989.

**Variables**	**Total**	**Urban *n*= 2,181**	**Non-urban *n*= 808**	***p*-value**
Age category, count (%)				0.04^*^^∧^
65–69	1,243	937 (43.0)	306 (37.9)	
70–74	1,036	740 (33.9)	296 (36.6)	
75–79	710	504 (23.1)	206 (25.5)	
Gender, count (%)				0.87^∧^
Female	1,587	1,156 (53.0)	431 (53.3)	
Race/ethnicity, count (%) *n*= 2,985				<0.01^*^^∧^
White, non-hispanic	2,556	1,772 (81.4)	784 (97.0)	
Black, non-hispanic	212	208 (9.6)	4 (0.5)	
Hispanic	81	72 (3.3)	9 (1.1)	
Other, non-hispanic	70	62 (2.9)	8 (1.0)	
Asian	66	63 (2.9)	3 (0.4)	
Marital status, count (%) *n* = 2,961				<0.01^*^^∧^
Married, live-in partner	1,974	1,403 (65.1)	571 (70.9)	
Divorced, separated, never married	609	497 (23.1)	112 (13.9)	
Widowed	378	256 (11.9)	122 (15.2)	
Education, count (%) *n* = 2,980				<0.01^*^^∧^
High school or less	336	180 (8.3)	156 (19.4)	
Some college	725	496 (22.8)	229 (28.4)	
Bachelor's degree	698	528 (24.3)	170 (21.1)	
Advanced degree	1,221	970 (44.6)	251 (31.1)	
Depression PROMIS T-Score, count (%) *n* = 2,985				0.59^∧^
>55 (increased depressive symptoms)	186	139 (6.4)	47 (5.8)	
≤55	2,799	2,041 (93.6)	758 (94.1)	
Self-reported vision, count (%)				0.79^∧^
Very good—excellent	2,005	1,460 (66.9)	545 (67.5)	
Poor—good	984	721 (33.1)	263 (32.6)	
Self-reported hearing, count (%)				0.04^*^^∧^
Good—excellent	2,640	1,942 (89.1)	698 (86.4)	
Poor—fair	348	238 (10.9)	110 (13.6)	
Physical function PROMIS T-Score, count (%)				0.56^∧^
<50 (worse physical function)	1,369	1,006 (46.1)	363 (44.9)	
≥50	1,620	1,175 (53.9)	445 (55.1)	
Social isolation PROMIS T-Score, *n* = 2,970, mean (SD)	42.9 (6.9)	42.9 (6.9)	42.9 (7.1)	0.77^~^
Social role PROMIS T-score, *n* = 2,922, mean (SD)	57.4 (6.9)	57.4 (6.9)	57.6 (6.8)	0.51^~^
Life satisfaction, *n* = 2,986, mean (SD)	3.9 (0.6)	3.9 (0.6)	3.9 (0.6)	0.56^~^
Miles driven per week, count (%) *n* = 2,918				<0.01^*^^∧^
0–49	696	550 (26.0)	146 (18.3)	
50–99	701	511 (24.1)	190 (23.8)	
100–150	794	539 (25.4)	255 (31.9)	
>150	727	519 (24.5)	208 (26.0)	

* *Significant <0.05*.

∧ *Chi-Squared*.

~ *T-Test*.

*^†^Fishers exact*.

Of the 2,989 participants 13.2% reported social isolation at baseline and the average T-score measures was 42.9, lower than average social isolation. A T-score of 50 is the mean and a T-score of 40 indicates one standard deviation below the mean corresponding with less social isolation. Variables from univariate testing as seen in Table 1 that were statistically significantly different between urban and non-urban settings were included in the model. Those were age (*p* = 0.04), race and ethnicity (*p* < 0.01), marital status (*p* < 0.01), education (*p* < 0.01), hearing (*p* = 0.04) and miles driven per week (*p* < 0.01). Depression, vision and hearing were significantly related to social isolation and also included in the full model.

Participants in urban areas appeared to be 28% less likely to be socially isolated than participants in non-urban areas in the unadjusted model (unadjusted rate ratio 0.72, 95% CI 0.43, 1.19) (Table 2). That gap narrowed to 21% in the adjusted model (adjusted rate ratio 0.79, 95% CI 0.46, 1.36) (Table 2). Neither rate was statistically significant. The adjusted model was globally significant in being able to meaningfully assess the relationship between geography and social isolation (χ^2^= 384.7, *p*-value < 0.01). Gender and vision were excluded as they did not contribute to the adjusted rate ratio.

**Table 2:** Geography status and rate of being socially isolated.

	**Unadjusted rate ratio (95% CI)**	**Adjusted rate ratio^**a**^ (95% CI)**
Urban	0.72 (0.43, 1.19)	0.79 (0.46, 1.36)

a *Adjusting for age, race/ethnicity, marital status, education, depression, hearing, physical function, miles driven per week*.

## Discussion

Social isolation is gaining increased attention as a predictor of poor health outcomes, now associated with increased risk of death and increased Medicare expenses, although geographic considerations have been lacking in this literature (7, 23). Disparities in health outcomes and health care access by geographic location have been documented extensively, as have the associations between isolation and health care utilization, suggesting that these complex relationships require further examination (8, 11). Assuming that social isolation is a modifiable risk factor for poor outcomes, more detailed information can support more tailored interventions.

These results align with a larger cross-sectional study in that social isolation was not significantly different between urban and non-urban areas (6). Cudjoe et al. (6) had sufficient power for its conclusions; however, it was limited by the cross-sectional nature of the data. In this analysis, we similarly saw no significant difference in social isolation between urban and non-urban areas. A subsequent study assessing social isolation by geography found increased social isolation in urban areas and found an association in rural areas with the potential to buffer the effects of social isolation: friends that can be relied upon, number of close family members, number of living children and grandchildren, number of friends (10). A major limitation in social isolation research is a lack of consensus in construct operationalization.

A strength of this study is the increased precision of the PROMIS Social Isolation SFv2.0 short form which is normed to US census data allowing for increased comparability. Panel data from three time periods provided more evidence for causality. A limitation was that the sample is not demographically diverse, although it was not designed to be a nationally representative sample. Alternate transportation use was excluded in this analysis as it was not operationalized to include rideshare but could be a potential confounder when assessing geographic differences (24). The suburban and rural categories, combined to represent non-urban areas were under-represented in this study compared to urban areas, potentially reducing study power for these results.

## Conclusions

The importance of social isolation is gaining more traction in research and it will be important to have a nuanced understanding of the experience in the geographically diverse areas of the US to ensure effective policies are implemented. Although geographic differences in social isolation were not statistically significant in this analysis, the evidence can build on the conversation in the literature regarding social isolation and rurality. This research was done prior to the COVID-19 pandemic; more research is needed to better understand the effect the pandemic may have had on social isolation, specifically older adults. Geographic location could potentially play a greater role in the setting of a pandemic and it needs further analysis.

## Data Availability Statement

Restrictions apply to the availability of these data. Data are available from the author with permission from the AAA Foundation for traffic safety and upon execution of a data use agreement.

## Ethics Statement

The studies involving human participants were reviewed and approved by IRB of the Columbia University Medical Center (IRB-AAAN9950). The patients/participants provided their written informed consent to participate in this study.

## Author Contributions

TM and LL: conceptualization, formal analysis, and writing—original draft preparation. TM, LL, GL, and DS: methodology. TM, DS, CD, MB, LM, DE, VJ, LH, and GL: data curation. TM, LL, DS, CD, MB, LM, DE, VJ, LH, and GL: writing—review and editing. TM: supervision. All authors have read and agreed to the published version of the manuscript.

## Funding

This work was supported by the AAA Foundation for Traffic Safety. This research was supported in part by Grant 1 R49 CE002096- 01 from the Centers for Disease Control and Prevention, National Center for Injury Prevention and Control to the Center for Injury Epidemiology and Prevention at Columbia University.

## Author Disclaimer

Its contents are solely the responsibility of the authors and do not necessarily represent the official views of the National Institutes of Health and Centers for Disease Control and Prevention.

## Conflict of Interest

The authors declare that the research was conducted in the absence of any commercial or financial relationships that could be construed as a potential conflict of interest.

## Publisher's Note

All claims expressed in this article are solely those of the authors and do not necessarily represent those of their affiliated organizations, or those of the publisher, the editors and the reviewers. Any product that may be evaluated in this article, or claim that may be made by its manufacturer, is not guaranteed or endorsed by the publisher.
